# Overexpression of *Galectin-7* in Mouse Epidermis Leads to Loss of Cell Junctions and Defective Skin Repair

**DOI:** 10.1371/journal.pone.0119031

**Published:** 2015-03-05

**Authors:** Gaëlle Gendronneau, Sadaf Sanii, Tien Dang, Frédérique Deshayes, Delphine Delacour, Evelyne Pichard, Tamara Advedissian, Sukhvinder S. Sidhu, Mireille Viguier, Thierry Magnaldo, Francoise Poirier

**Affiliations:** 1 Institut Jacques Monod, UMR CNRS 7592, Paris-Diderot University, Paris, France; 2 IRCAN, CNRS-Inserm-UNS UMR 7284, U 1081, Nice, France; CNRS-University of Toulouse, FRANCE

## Abstract

**Background:**

The proteins of the galectin family are implicated in many cellular processes, including cell interactions, polarity, intracellular trafficking, and signal transduction. In human and mouse, galectin-7 is almost exclusively expressed in stratified epithelia, notably in the epidermis. *Galectin-7* expression is also altered in several human tumors of epithelial origin. This study aimed at dissecting the consequences of *galectin-7* overexpression on epidermis structure and functions *in vivo*.

**Methods:**

We established transgenic mice specifically overexpressing *galectin-7* in the basal epidermal keratinocytes and analyzed the consequences on untreated skin and after UVB irradiation or mechanical injury.

**Results:**

The intercellular cohesion of the epidermis is impaired in transgenic animals, with gaps developing between adjacent keratinocytes, associated with loss of adherens junctions. The epidermal architecture is aberrant with perturbations in the multilayered cellular organisation of the tissue, and structural defects in the basement membrane. These transgenic animals displayed a reduced re-epithelialisation potential following superficial wound, due to a defective collective migration of keratinocytes. Finally, a single mild dose of UVB induced an abnormal apoptotic response in the transgenic epidermis.

**Conclusion:**

These results indicate that an excess of galectin-7 leads to a destabilisation of adherens junctions associated with defects in epidermal repair. As this phenotype shares similarities with that of *galectin-7* null mutant mice, we conclude that a critical level of this protein is required for maintaining proper epidermal homeostasis. This study brings new insight into the mode of action of galectins in normal and pathological situations.

## Introduction

The epidermis, the uppermost compartment of the skin, is in direct contact with the environment. Its major function is to provide the first line of body defense against environmental insults such as pathogens, UV irradiation, chemical and mechanical stresses. The epidermis is mainly composed of stratified layers of keratinocytes, which are constantly being regenerated from the underlying pool of dividing cells. These cells undergo complex biochemical and structural modifications from the deepest basal layer where they proliferate to the superficial layer where they are terminally differentiated into corneocytes [1,2]. The fine equilibrium between epidermal cell renewal and differentiation is crucial to establish a well formed and functional tissue. Abnormal cell proliferation, differentiation or cohesion in the epidermis result in pathological effects and severe skin diseases in humans.

Galectin-7 (14kDa) is one of the 15 members of the mammalian galectin family of soluble lectins, all of which share a homologous carbohydrate recognition domain that typically binds beta-galactoside residues [3,4,5]. In mouse and human, galectin-7 was initially discovered as a specific marker of stratified epithelia, including the epidermis [6,7], and indeed during mouse embryogenesis, *galectin-7* expression starts at the onset of epidermal stratification [8]. In adult mice, galectin-7 is found in all living epidermal cell layers with particularly strong expression in differentiated keratinocytes [9]. In humans, altered expression of *galectin-7* has been reported in tumors of epithelial origin and in cancer cell lines [10]. In particular, *galectin-7* gene is upregulated in pharynx, breast and ovarian cancers [11,12,13].

Although the exact roles of galectin-7 in normal or pathological epithelial tissues is not yet well understood, characterization of *galectin-7* deficient mice has uncovered a requirement for this protein in the maintenance of epidermal homeostasis. The skin repair properties of *galectin-7* deficient mice are severely affected as revealed by a delay in wound closure after mechanical injury and by an abnormal apoptotic response after UVB exposure [14]. These results are in accordance with earlier studies implicating *galectin-7* in re-epithelialisation of wounded cornea *in vitro* [15], and also in apoptosis of sunburn keratinocytes [16]. It has also been reported that the ectopic expression of *galectin-7* can render some tumor cell lines more susceptible to chemically induced apoptosis [17].

The goal of the present study was to gain further insight into the role of galectin-7 in the epidermis by overexpressing this gene in the proliferative, undifferentiated keratinocytes of the basal epidermal layer.

## Materials and Methods

### Transgenic mouse lines and genotyping

**Fig 1:**
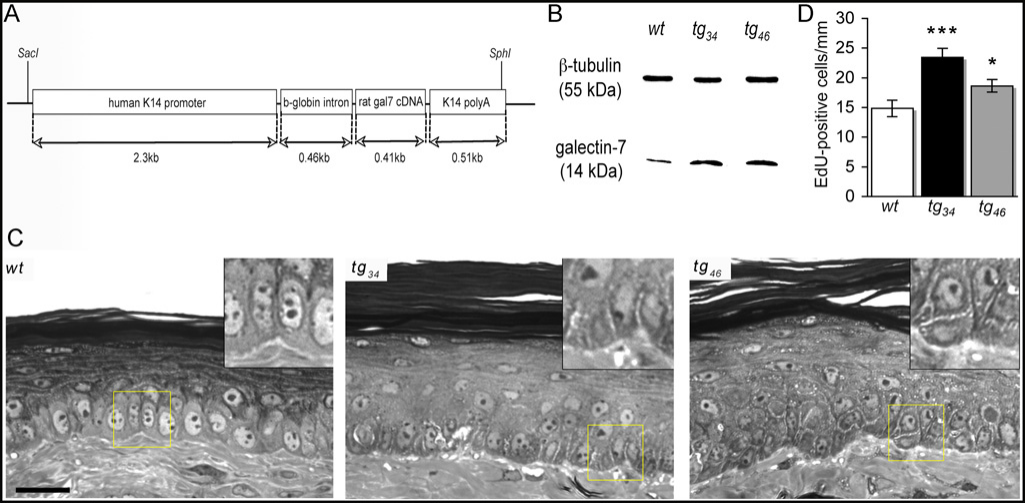
Skin abnormalities following *galectin-7* overexpression in basal epidermal keratinocytes. (A) *Galectin-7* expression vector. The rat *galectin-7* cDNA was inserted into the *K14* expression plasmid comprising the human *K14* promoter, the rabbit *ß-globin* intron and the *K14* polyA sequence [9]. Two independent transgenic lines were obtained. (B) Western blot analysis. Skin protein extracts from wild-type (*wt*), *tg*_*34*_ and *tg*_*46*_ transgenic mice were used. Galectin-7 (14 kDa) and control β-tubulin (55 kDa) proteins were detected. (C) Representative semi-thin sections of *wt*, *tg*_*34*_ and *tg*_*46*_ tail skin are shown. Epidermal thickening, cell disorganization and loose basal lamina are observed in *tg*_*34*_ and *tg*_*46*_ skin compared to *wt* tissue. Scale bar: 20μm. (D) Quantification of cell proliferation. A significantly higher number of EdU-positive keratinocytes per mm of linear epidermis was detected in *tg*_*34*_ and *tg*_*46*_ samples compared to *wt*. A total of 34 mice were used for these measurements (N*wt* = 12; N*tg*_*34*_ = 10; N*tg*_*46*_ = 12). Each bar represents the mean value ± sem. Statistical differences between *wt* and transgenic animals are noted *p<0.05, ***p<0.001.

The pGEM3Z-K14 vector (kindly provided by E. Fuchs) [18] was used to clone the rat *galectin-7* cDNA [9] downstream of the basal cell specific *keratin14* (*K14*) promoter. C57Bl/6; DBA/2 ovocytes were microinjected with a Sac1/Sph1 fragment containing the construct (see Fig. 1 and [18]). Founder animals carrying the transgene were mated with C57Bl/6 individuals and two independent *K14-galectin-7* transgenic lines, named *tg*_*34*_ and *tg*_*46*_, were established after eight successive backcrosses on the C57Bl/6 background. For genotyping, genomic DNA was obtained from tail snips and a 450 bp fragment of the transgene was amplified by polymerase chain reaction using the following specific primers: 5’-gctggcgtggaaatattcttattgg-3’ and 5’-GCCGCATAGCAGGTTTACATGG-3’. All experiments were performed on 2 months-old heterozygous transgenic female mice. Animals were kept in a specific pathogen-free (SPF) animal house facility and experiments were performed in accordance with European and French Agricultural Ministry guidelines for the care and use of laboratory animals (Council directives 2889 and 86/609/EEC). These experiments and protocols were approved by the institutional ethics committee “The Animal Experimentation Ethical Committee Buffon” (CEEA-40) under the reference CEB-004–2011.

### Western blot analysis on mouse tissue

Protein extraction and Western blot analysis were done as previously described [14]. Galectin-7 protein was detected using the rabbit polyclonal anti-galectin-7 antibody (1:3000; [7]) and horseradish peroxidase-conjugated goat anti-rabbit antibody (1:10000, GE Healthcare NA934, Little Chalfont, Buckinghamshire, United Kingdom). ß-tubulin protein was detected with a mouse monoclonal anti-ß-tubulin antibody (1:8000, GE Healthcare N357) and horseradish peroxidase-conjugated goat anti-mouse antibody (1:15000, A9044, Sigma-Aldrich, St. Louis, MO). Signals were revealed using the ECL Plus detection system (GE Healthcare RPN2132).

### Histology, measurement of epidermal thickness and immunostainings

For paraffin sections, tail or shaved back skin samples were fixed overnight, at 4°C, in PBS containing 4% paraformaldehyde, then dehydrated in a graduated series of ethanol up to 95% and cleared for 3h in isopropanol. Samples were next incubated for 36h at 60°C in paraffin (Paraplast) before final embedding. For cryosections, tail skin samples were directly embedded in OCT (Euromedex, France) and frozen on dry ice before storing at -80°C. Five-micron thick paraffin sections or ten-micron thick cryosections were prepared for immunofluorescence staining using standard protocols.

Ten measurements performed on seperate hematoxylin eosin stained sections were used to calculate the epidermal thickness of each individual. These measurements were done on three animals per genotype.

Primary antibodies: rabbit polyclonal anti-galectin-7 antibody (1:1000, [7]; rabbit polyclonal anti-keratin14 antibody (1:1000, Covance, Emeryville, CA, PRB 155P); mouse monoclonal anti-keratin10 (1:100, Covance PRB 159S); mouse monoclonal antibody specific for desmogleins 1 and 2 (1:50, 03–61002; American Research Products, Belmont, MA); rabbit polyclonal anti-plakoglobin antibody (1:25, C2069–60A; U.S. Biological, Swampscott, MA); mouse monoclonal anti-E-cadherin antibody (1:100, 610181; BD Biosciences Dickinson, San Jose, CA); rabbit polyclonal anti-ß-catenin antibody (1:200, C2206, Sigma-Aldrich); rabbit polyclonal anti-laminin antibody (1:200, L9393, Sigma-Aldrich); mouse monoclonal anti-cortactin (1:200, 05–180 Upstate, Millipore).

Secondary antibodies: Alexa488-conjugated goat anti-rabbit antibody (1:200, A11008; Invitrogen, Cergy Pontoise, France); Alexa568-conjugated goat anti-mouse antibody (1:200, A11004; Invitrogen

Nuclei were stained with Hoechst33342 (H3570, Invitrogen) and confocal analysis was performed with a Leica SP5 microscope.

### Ultrastructural analysis

Samples of tail skin or shaved back skin were immersion-fixed in 1.6% glutaraldehyde in Milloning buffer (0.1M Na_2_HPO_4_, 0.1M NaHPO_4_, pH7.3), for 2 days at 4°C, and subsequently postfixed in 2% osmium for 2h at room temperature. Standard procedures for dehydration and embedding in Epon-Araldite (Electron Microscopy Sciences) were used. Observations were performed using a Tecnai 12 transmission electron microscope. Two mice of each genotype were analysed.

### Quantification of proliferative cells

Mice were injected intraperitoneally with 100μl of a 300μg/ml solution of 5-ethylnyl-2′-deoxyuridin (EdU) solution in PBS. Six hours later, the animals were sacrificed and tail skin samples were fixed in 4% paraformaldehyde before embedding in paraffin. EdU detection was based on a reaction covalently linking alkyne containing EdU and Alexa Fluor dye, according to the manufacturer’s recommendations (C10337, Invitrogen). Quantification of EdU-positive cells was restricted to interfollicular regions.

### *In vivo* wound-healing experiments

Mice were anesthetized by intraperitoneal injection of a mixture of ketamine-xylazine (50mg/kg and 5mg/kg, respectively), and a superficial scratch was made with a sterile blood lancet (Assistant, Sondheim, Germany) along the dorsal side of the tail. Animals were sacrificed by cervical dislocation at 18h after injury. Injured tail segments (0.5 cm) were processed for paraffin embedding or frozen in OCT for cryosectioning. The distance between wound margins was measured on hematoxylin and eosin stained transverse sections. All results are expressed as a mean of at least three independent values per individual, taken at least 20 μm deep in the paraffin block.

### *In vivo* UVB irradiation and detection of apoptotic cells

UVB irradiation experiments were performed as previously described [14]. Briefly, mice were anesthetized and the back hair of mice was removed with a depilatory cream. Five days later, the posterior region of the depilated zone was exposed to a single dose of 2000J/m^2^ of UVB, again under general anaesthesia. The unirradiated anterior region served as a control.

Apoptotic sunburn cells were identified on paraffin sections stained with hematoxylin-eosin using the following criteria: membrane shrinkage, dark condensed nucleus and eosinophilic cytoplasm [14,19]. Alternatively, Terminal Deoxynucleotidyl Transferase Nick End Labeling (TUNEL) assay was carried out using the *In Situ Cell Death Detection* Kit (Roche). For each animal, quantification was carried out along two 1cm long sections, spaced 200 μm apart. This analysis was restricted to interfollicular regions. Results are expressed as a number of apoptotic cells per cm length of epidermis.

### Co-immunoprecipitation

HaCat cells [20] were grown in DMEM (Invitrogen, Cergy-Pontoise, France) containing 10% fetal calf serum, supplemented with antibiotics, and maintained at 37°C in 5% CO2 atmosphere. Normal human epidermal keratinocytes (NHEK, Catalogue No. C-12001 purchased from PromoCell GmbH, Heidelberg, Germany) were maintained at low 0.06mM CaCl2 (C-34005) concentration in keratinocyte growth medium 2 (C-20011) containing SupplementMix (C-39016), all from PromoCell GmbH. Whole cell extracts were prepared from confluent 10cm tissue culture dishes, using 200μl of 25mM TrisHCl buffer pH7.5 containing 100mM NaCl, 1mM EDTA (E5134- Sigma-Aldrich, St. Louis, MO) 1mM EGTA (E3889-Sigma-Aldrich), 0.5% NP40, 1% TX100, 1:100 protease inhibitor cocktail (P8340, Sigma-Aldrich). The nuclear fraction was eliminated by centrifugation and the supernatant was incubated in the presence of 60μl of protein A-sepharose beads (P9424-Sigma-Aldrich) for 1h, under gentle agitation. The beads were eliminated by centrifugation at 5000rpm for 5min at 4°C. Then the primary antibody (rabbit anti human galectin-7 antibody—ab10482-Abcam, Paris, France—or mouse anti human E-cadherin—610181-BD Biosciences, 38800 Le Pont de Claix, France or control IgG- 349050-BD Biosciences, 38800 Le Pont de Claix, France) was added to the supernatant and incubated overnight, at 4°C. Sixty μl of protein A-sepharose beads were added, incubated for 3h before washing twice in 500μl PBS containing 0.1% NP40 and twice in PBS. Samples were analysed on a 12% acrylamide gel. Western blot analysis was performed using the rabbit anti-human galectin-7, mouse anti-E-cadherin or rabbit anti-keratin16 (ab53117-abcam Paris, France) primary antibodies, and visualized using a anti-rabbit (NA934V-GE Healthcare, Little Chalfont, Buckinghamshire, United Kingdom) or anti-mouse (A9044, Sigma-Aldrich) horseradish peroxidase-conjugated secondary antibody. Signals were revealed using ECL Plus detection system (RPN2132-GE Healthcare).

### Statistical analysis

The data were expressed as the mean value± sem. The non-parametric Mann-Whitney U-test was used for comparisons (Software StatEL).

## Results

### Overexpression of *galectin-7* in skin

In order to overexpress galectin 7 in the basal epidermal layer, the rat *galectin-7* cDNA [9] was cloned downstream of the basal cell specific *keratin14* (*K14*) promoter, and used to establish two independent *K14-galectin-7* transgenic mouse lines (Fig. 1A), named *tg*_*34*_ and *tg*_*46*,_ on the C57Bl/6 background. Western blot analysis performed on skin extracts indicated a 3.8- and a 2-fold increase in the amount of the protein in *tg*_*34*_ and *tg*_*46*_ samples respectively, compared to *wt* samples (Fig. 1B) (This was also documented by immunostaining with anti-galectin-7 antibody; see below).

The viability of transgenic animals was similar to that of *wt* littermates in SPF animal housing conditions and they did not display any obvious skin, hair or whisker phenotype.

Comparison of semi-thin sections of tail skin samples readily showed that, in *tg*_*34*_ and *tg*_*46*_ transgenic tissue, the superficial cornified layers were well developed and similar to that of *wt* skin; in contrast, the underlying epidermis was affected (Fig. 1C). The overall thickness of the epidermal layers was larger in the transgenics (*tg*_*34*_ 50.46+/-0.23μm, n = 3, p<0.00001; *tg*_*46*_ 41.26+/-4.09μm, n = 3, p<0.0002) than in the control *wt* epidermis (35.09 +/-0.46μm, n = 3). This was associated with a higher number of EdU-positive cells in both *tg*_*34*_ (1.6-fold increase relative to *wt*, p<0.001) and *tg*_*46*_ (1.3-fold increase relative to *wt*, p<0.05) epidermis, indicating a state of moderate, but significant hyperproliferation (Fig. 1D).

**Fig 2:**
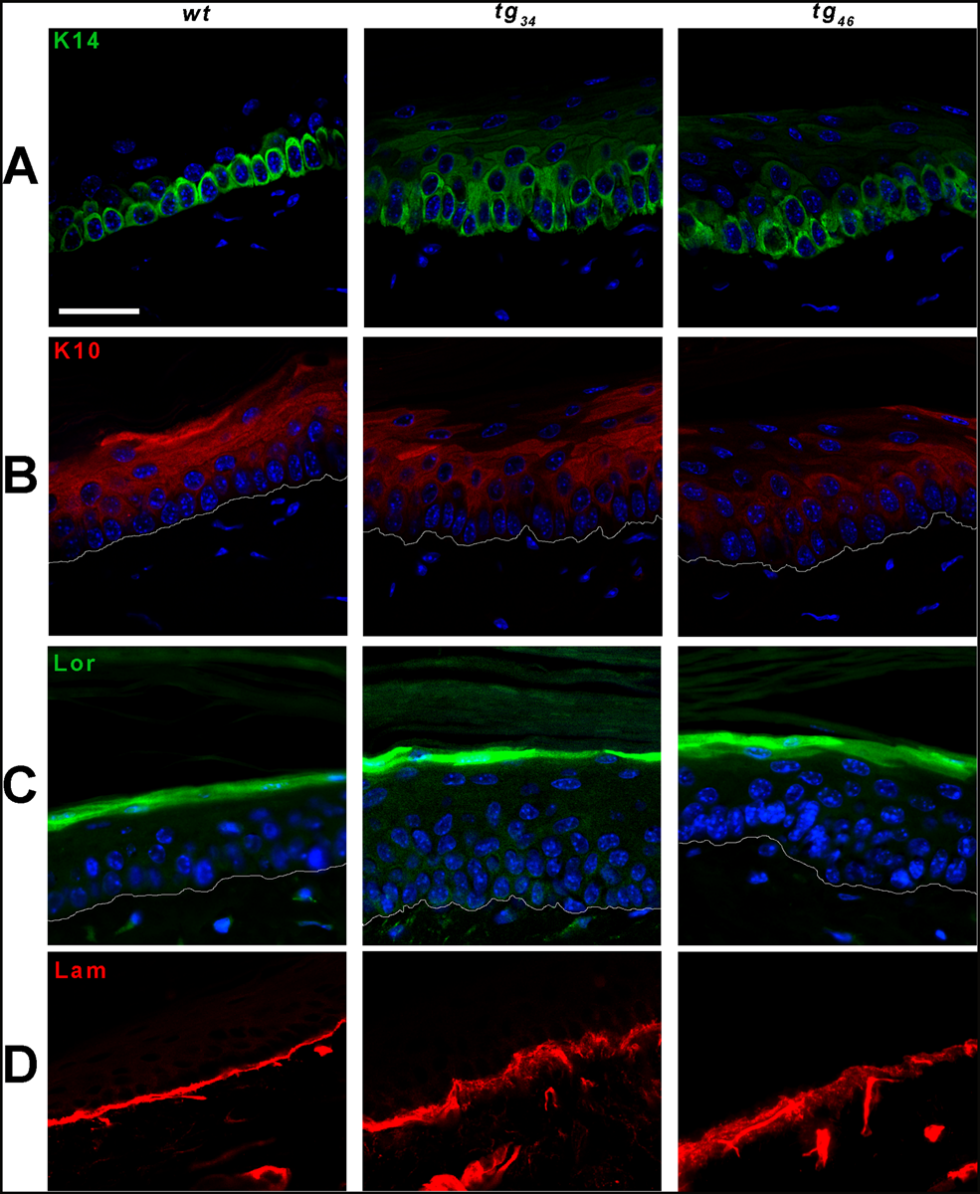
Defects in epidermal markers in *tg*_*34*_ and *tg*_*46*_ epidermis. Representative immunofluorescent stainings of keratin14 (K14), specific for basal cells (panel A), keratin10 (K10), specific for suprabasal cells (panel B), and loricrin, specific for terminally differentiated cells (panel C), were performed on paraffin sections of tail skin and analysed by confocal microscopy. Abnormal K14 staining is observed in cells above the basal layer, while K10 staining appears less intense in suprabasal cells of transgenic samples compared to *wt*. Three to five mice were used for each genotype. White lines delineate the dermo-epidermal junction. Scale bar: 20μm.

The structure of the tissue was also perturbed in the transgenic epidermis. In *wt* skin, basal keratinocytes formed a uniform palissadic row of cells lying above the dermis, all displaying a large nucleus and a characteristic cuboidal shape. In the epidermis of both transgenic lines, basal cells were very uneven in size, their shape was irregular, and their nuclei appeared smaller than in *wt* epidermis (Fig. 1C). These morphological differences were corroborated by abnormalities in the expression patterns of differentiation markers. The compartment of K14-positive keratinocytes was broader, encompassing several cell layers in the epidermis of transgenic individuals (although the effect is more pronounced in *tg*_*34*_ than in *tg*_*46*_), while mainly restricted to a single basal cell layer in *wt* skin (Fig. 2A). The suprabasal keratin10 (K10) staining appeared weaker in some regions of transgenic epidermis, compared to *wt* tissue, and some cells were even K10-negative in suprabasal layers (Fig. 2B). The border between basal (K14-positive) and suprabasal (K10-positive) compartments was less sharply delineated in transgenic epidermis than in *wt* skin. Despite these early perturbations in the keratinocyte differentiation program, the expression of the terminal differentiation marker, loricrin, appeared normal in transgenic tissues (Fig. 2C), which was consistent with the well developed cornified layers (see Fig. 1C).

Histological sections also showed irregularities located at the dermo-epidermal junction in both *tg*_*34*_ and *tg*_*46*_ transgenic skin (Fig. 1C). Immunostaining with an anti-laminin antibody revealed defects at the level of the basement membrane in transgenic animals: the signal appeared broader, irregular, and ragged in *tg*_*34*_ and *tg*_*46*_ samples, whereas a regular thin line was observed in *wt* skin (Fig. 2D).

In summary, despite the apparent normal global appearance of the transgenic mice, overexpression of *galectin-7* leads to structural abnormalities of the epidermis.

### Loss of intercellular cohesion in *galectin-7* overexpressing epidermis

**Fig 3:**
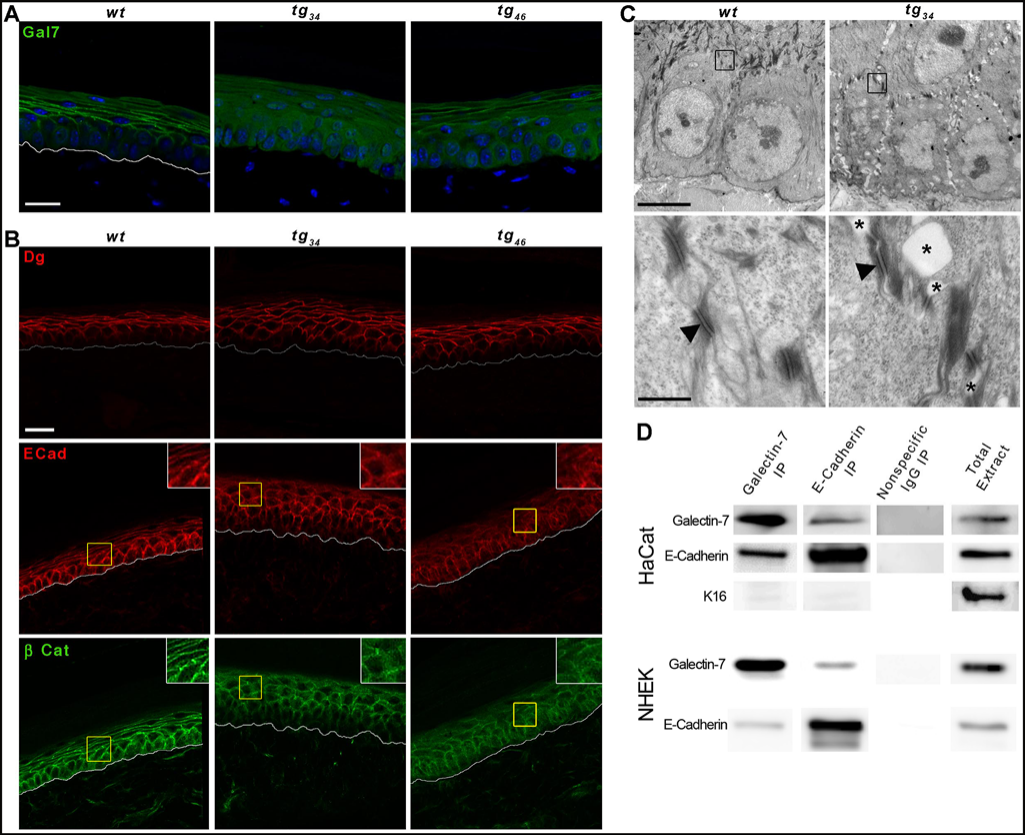
Aberrant intercellular junctions in *tg*_*34*_ and *tg*_*46*_ epidermis. (A-B) Confocal analysis of immunofluorescent stainings performed on paraffin sections of *wt*, *tg*_*34*_ and *tg*_*46*_ tail skin. Three to five mice were used for each genotype. White lines delineate the dermo-epidermal junction. Scale bar: 20μm. (A) Galectin-7 profile. Enhanced galectin-7 staining is detectable in both basal and suprabasal cells of *tg*_*34*_ and *tg*_*46*_ epidermis compared to *wt*. (B) Intercellular junctions. Desmosomes are labeled by anti-desmoglein (Dg) staining which appears similar in *wt*, *tg*_*34*_ and *tg*_*46*_ epidermis. Adherens junctions are marked by anti-E-cadherin (Ecad) and anti-ß-catenin (ß-cat) staining. A more diffuse signal was observed in *tg*_*34*_ and *tg*_*46*_ samples compared to the *wt* control. (C) Transmission electron microscopy analysis. Upon low magnification, large regions exhibiting aberrant widening of intracellular spaces are observed in transgenic epidermis (upper panels). Higher magnification analysis revealed the presence of large intercellular spaces in transgenic tissue (asterisks) compared to *wt* (lower panels). There is a loss of adherens junctions while desmosomes (black arrowheads) are preserved in transgenic epidermis. Two mice were used for each genotype. Scale bars: upper panels: 5μm; lower panels: 0.5μm. (D) Coimmunoprecipitation experiment on cell lysates of HaCat (top panels) or primary normal human epidermal keratinocytes (NHEK, passage 2; bottom panels), using anti-galectin-7 and anti-E-cadherin antibodies. Whole cell extracts and normal IgG immunoprecipitations were used as positive and negative controls, respectively.

In mouse skin, *galectin-7* is expressed in epidermal keratinocytes, and not in dermal fibroblasts [14]. In *wt* epidermis, galectin-7 protein is very weakly but uniformly detected in the cytoplasm of basal keratinocytes, while in suprabasal cells it is more abundant and mainly associated with plasma membranes (Fig. 3A). In transgenic animals, the basal keratinocytes (where the *K14* promoter is known to be active) display an intense galectin-7 signal in the cytoplasm. There is also a concomittant increase in galectin-7 protein amount in the suprabasal layers, presumably reflecting the stability of the protein during keratinocyte differentiation. However, rather than being predominantly at the plasma membrane, this strong signal is uniformly distributed throughout the cytoplasm of suprabasal keratinocytes.

We next studied adhesion structures by analysing the distribution of desmogleins 1/2 which are epidermal desmosomal components, as well as the distribution of E-cadherin and ß-catenin, two major adherens junction proteins. The desmoglein staining was equally regular, sharp and intense along the cell membranes of *wt* and transgenic keratinocytes, suggesting that desmosomes were unaffected (Fig. 3B). As expected, the E-cadherin and ß-catenin stainings were strictly overlapping but their distribution was altered in the transgenic skin samples (both *tg*_*34*_ and *tg*_*46*_) compared to the controls. In transgenic epidermis, E-cadherin/ß-catenin immunostaining appeared less regular along the cell membranes (Fig. 3B); moreover, some aberrant low level of cytoplasmic signal was also detected, indicating that a fraction of E-cadherin and ß-catenin was no longer restricted to the cell membrane. These observations suggested that adherens junctions may be altered in transgenic skin. This was confirmed at the ultrastructural level by transmission electron microscopy (Fig. 3C). Instead of the tight intercellular contacts characteristic of *wt* epidermal cells, the keratinocytes of transgenic animals showed a distinct defect in cohesion, in that the cells were loosely attached to each other. Upon higher magnification, the desmosomes appeared morphologically normal, as expected based on the desmoglein immunostaining. In contrast, large intercellular gaps were found between adjacent desmosomes, suggesting a weakening of adherens junctions in transgenic skin, consistent with the diffuse E-cadherin/ß-catenin staining (Fig. 3C).

Taken together, the results from confocal and electron microscopy establish that overexpression of *galectin-7* provokes defects in adherens junctions and impairment of the overall cohesion of the epidermis (Note that this was also observed on transgenic back skin samples, see S1 Fig.).

Since galectin-7 is normally located along the plasma membranes (Fig. 3A), we next examined whether galectin-7 might be interacting with E-cadherin, the main constituent of adherens junctions. Using extracts prepared from confluent cultures of either the HaCat keratinocyte cell line or primary human epidermal keratinocytes, we found that galectin-7 and E-cadherin could be reciprocally co-immunoprecipitated (Fig. 3D). This result suggests that galectin-7 may indeed play a role in adherens junctions formation or stability.

### Delayed wound closure in *K14*-*galectin7* transgenic mice

These structural defects prompted us to explore potential consequences of galectin-7 overexpression on skin repair after environmental stress. Firstly, we made a superficial scratch along the length of the tail in order to study wound healing. By leaving the dermis mostly intact, such a scratch minimizes the potential contribution from dermis contraction during the wound-healing process. As previously described, each margin of the wounded epidermis then starts moving to fill the gap and this process of collective migration lasts for a few hours following the scratch. Re-epithelialisation is normally completed in less than two days [14].

**Fig 4:**
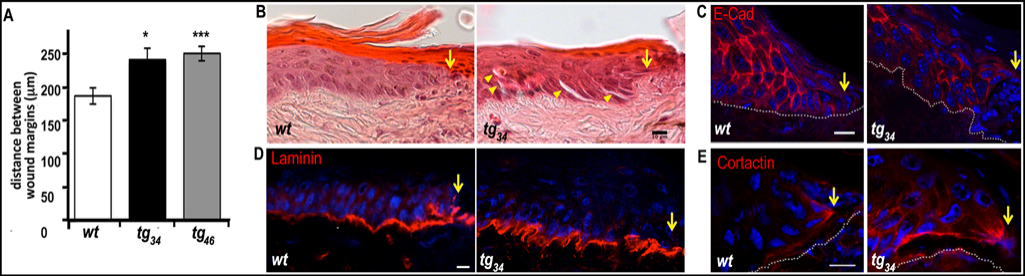
Delayed wound closure in transgenic mice. Superficial scratches were made along the sagittal axis of the tail. (A) Wound closure measurement. The distance between the two wound margins was determined 18h after injury. Results were calculated as a mean of at least three independent measurements per animal. A total of 49 mice were used for these experiments (N*wt* = 20; N*tg*_*34*_ = 14; N*tg*_*46*_ = 15). Each bar represents the mean value ± sem. Statistical differences between *wt* and transgenic animals are noted *p<0.05, ***p<0.001. (B) Histology. All pictures are focused on the “left” side of the migrating wound margin (yellow arrows). Transverse tail sections of the wound site stained with hematoxylin and eosin revealed abnormal migrating epithelial tongues in transgenic tissue compared to control, including signs of microblistering (yellow arrowheads). Scale bar: 10μm. (C, D, E) Confocal analysis. Immunostainings are performed either on paraffin sections for anti-E-cadherin Ab (C), or on cryosections for anti-laminin (D) or anti-cortactin (E) Abs. White lines delineate the dermo-epidermal junction. Scale bar: 10μm.

We found that, at 18h post injury, the distance between the wound margins was systematically larger in the transgenic animals (29% larger in *tg*_*34*_ *vs wt*, p<0.05; 34% larger in *tg*_*46*_ *vs wt*, p<0.001) (Fig. 4A). Overexpression of *galectin-7* in epidermis thus leads to slowing of wound closure.

Histological examination revealed that the migrating epidermal tongues were well-organized in *wt* epidermis, with the entire epithelium remaining tightly cohesive and a few cells adopting an elongated shape at the front (Fig. 4B). In contrast, there was a global perturbation of cell shape, size and organisation in the migrating keratinocytes of the *tg*_*34*_ (Fig. 4B) and *tg*_*46*_ (data not shown) healing epidermis, even distal to the wound; there were intercellular gaps between adjacent keratinocytes, and signs of microblistering were occasionally detected (Fig. 4B). In support of these observations, E-cadherin staining was much fainter and more diffuse in the transgenic migrating cells than in the *wt* (Fig. 4C). Moreover, laminin staining revealed the formation of an undulating and ragged basement membrane extending a long distance from the wound, whereas in the *wt*, this intense laminin staining was found only in the region just adjacent to the wound (Fig. 4D).

We also analysed the localization of cortactin (Fig. 4E), a marker of membrane ruffles which are formed at the leading edge of the cell during migration [21]. In the *wt* tissue, a discrete accumulation of cortactin was found at the advanced tip of only one (or very few) leader keratinocyte(s); the other migrating cells did not display any detectable signal. In contrast, several keratinocytes displayed a polarized cortactin signal in the transgenic wound edge, moreover aberrant cortactin staining was also present in keratinocytes located behind the leading edge, indicating a disorganisation of the migrating tongue.

In summary, we have found that the defects in cell adhesion and basement membrane previously observed in resting epidermis of transgenic animals (Figs. 2 and 3) were exacerbated after injury (Fig. 4). This resulted in defects in the process of collective migration which provoked a delay of wound closure.

### Abnormal skin response to UVB injury in *K14*-*galectin7* transgenic mice

**Fig 5:**
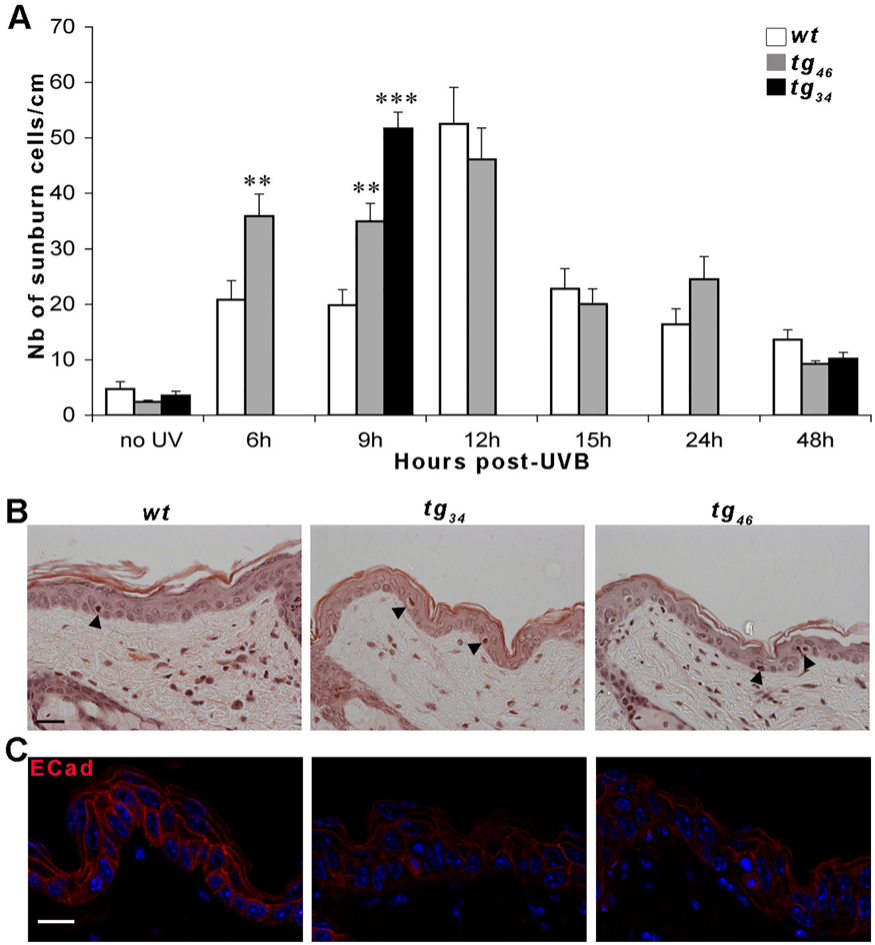
Aberrant epidermal response in transgenic mice after UVB irradiation. Mice were depilated on their back and irradiated with a single dose of UVB (2000J/m^2^). (A) Kinetic of apoptotic response. The number of sunburn cells per cm of epidermis was determined in unirradiated depilated skin (no UV) and in irradiated skin between 6h and 48h post-UVB exposure. A total of 61 mice were used for these experiments (N*wt* = 25; N*tg*_*34*_ = 11; N*tg*_*46*_ = 25) i.e. 3 to 4 mice per genotype and per time point. Each bar represents the mean value ± sem. Statistical differences between *wt* and transgenic animals are noted **p<0.01 and ***p<0.001. (B) Histology. Representative hematoxylin-eosin sections of irradiated skin samples at 9h post-UVB. Apoptotic sunburn cells are indicated by black arrowheads. (C) Confocal analysis of E-Cadherin immunostaining on skin samples at 9h post-UVB. The signal is very weak and diffuse in *tg*_*34*_ and *tg*_*46*_ tissues compared to the *wt*. Three mice were used for each genotype. Scale bar: 20μm.

As a second test of skin repair in response to environmental stress, we investigated the response of the transgenic epidermis following UVB exposure. We had previously designed a protocol to follow the effect of moderate irradiation of UVB *in vivo* [14]. Briefly, areas of depilated back skin were exposed to a single light dose of UVB (2000 J/m^2^) and the extent of apoptosis was quantified by monitoring sunburn cell formation over a period of 48h (Fig. 5A and B). Apoptotic sunburn cells were identified on paraffin sections stained with hematoxylin-eosin using the following criteria: membrane shrinkage, dark condensed nucleus and eosinophilic cytoplasm [19] (Fig. 5B).

There was no difference in the basal levels of apoptotic cells between *wt* and *tg*_*34*_ or *tg*_*46*_ transgenic epidermis prior to irradiation, ruling out an impact of *galectin-7* overexpression on the baseline level of apoptosis *in vivo* (see “no UV” in Fig. 5A). The maximal levels of the apoptotic response occurred at 12h post-UVB and was of equivalent intensity (about 50 sunburn cells/cm) in both transgenic and *wt* epidermis. However, the kinetics differed between the two sets of mice. In the case of *wt* skin, the phase of intense apoptotic response (>30 sunburn cells/cm) was limited to a narrow time frame around 12h post-UVB whereas, in the transgenic *tg*_*46*_ epidermis, the intense response spanned a much longer period, from 6 to 12h post UVB. Indeed, there were 1.8-fold more apoptotic cells in *tg*_*46*_ epidermis as in *wt* epidermis at both 6h and at 9h post-UVB time points. This was confirmed by a TUNEL assay at 6h post-UVB (*tg*_*46*_ 21.7+/-7.9 TUNEL positive cells/cm, n = 3; *wt* 4.7+/-2.5 TUNEL positive cells/cm, n = 3; p<0.00001). The second transgenic line *tg*_*34*_ behaved similarly, with a 2.6-fold increase in the number of sunburn cells compared to *wt*, at 9h post-UVB (Fig. 5A).

Finally, we looked at the E-cadherin distribution consecutive to UVB exposure. The signal was very faint and diffuse in irradiated transgenic epidermis, indicating a low level of expression and a delocalisation of the adherens junction marker in comparison to the *wt* irradiated tissue (Fig. 5C). In summary, the apoptotic response to UVB exposure started earlier and lasted longer in *galectin-7* overexpressing transgenic animals than in the *wt*.

## Discussion

We describe here the characterization of two transgenic mouse lines overexpressing *galectin-7* in basal keratinocytes of the skin. The epidermal architecture of these transgenic animals is perturbed, with defects in the multilayered cellular organisation of the tissue. One of the most prominant features is the existence of large intercellular clefts between adjacent keratinocytes which results from the disruption of adherens junctions. In addition to this loss in tissue cohesion, we also observed that the basement membrane at the dermis-epidermis interface is disorganized.

These structural defects are associated with perturbations in the early stages of keratinocyte differentiation. In normal epidermis, only the basal keratinocytes in contact with the basement membrane express the K14/K5 pair of keratins, and a programmed switch to the K10/K1 pair occurs when these cells move up towards the surface as they undergo terminal differentiation [1]. In transgenic epidermis, we found that the compartment of K14-expressing cells is expanded to encompass several cell layers thus revealing defects in the initial stepwise program of differentiation. Interestingly, despite these distinct abnormalities, terminal keratinocyte differentiation still takes place with the formation of well-developed cornified layers.

Other studies examining the importance of adherens junction proteins in mouse postnatal skin have shown that conditional ablation of E-cadherin leads to a loss of adherens junctions and a concomittant disorganisation of the basement membrane [22]. Lack of alpha-catenin, an adaptor protein which links the adherens junctions to the actin cytoskeleton, also provokes defects in adherens junctions which are associated with a state of hyperproliferation and epidermal acanthosis [23]. Given the similarities between these mutants and the transgenic animals described here, we conclude that the various aspects of the *K14-galectin-7* phenotype also derive from this primary defect in adherens junctions. That galectin-7 itself may play a role in adherens junctions is suggested by its normal distribution along the plasma membrane of the keratinocytes in *wt* epidermis, and also by the results of co-immmunoprecipitation experiments which support a direct or indirect interaction between galectin-7 and E-cadherin in keratinocytes. Therefore, the most parsimonious interpretation of our data is that galectin-7 is normally involved in the formation and/or maintenance of adherens junctions and that these dynamic membrane structures can be destabilised by an excess of galectin-7. Interestingly, an interaction between another galectin, galectin-3, and N-cadherin has recently been described in a mouse mammary tumor cell line and this interaction can also modulate the dynamics of cell junctions [24]. A recent report also highlights the potential importance of galectin-7 in intercellular adhesion [25].

The cohesion defects observed in *galectin-7* overexpressing epidermis have no obvious physiological consequences in adult mice. In contrast, the epidermal response to environmental insults is defective in these transgenic animals. First, after a superficial scratch, there was a delay in the timing of wound closure. During re-epithelialisation, the « collective migration » of cells located at each edge of the wound, depends on sustained cellular interactions, notably through adherens junctions [26]. Based on our results, we propose that a main factor contributing to the delay in wound closure in transgenic animals is the loosening of adherens junctions.

In a second series of experiments, we exposed the mice to a single mild dose of UVB irradiation which normally induces a discrete peak of keratinocyte apoptosis, 12h later. We found that, in transgenic epidermis, the apoptotic response began earlier (already intense at the 6h timepoint), and lasted longer. That an excess of galectin-7 induced more keratinocyte apoptosis is consistent with earlier observations [16,17,27,28], notably the fact that galectin-7 has been described as a p53 target [27]. In the transgenic epidermis, we speculate that the keratinocytes overexpressing *galectin-7* are more susceptible to anoikis due to the defects in tissue cohesion and in basement membrane organisation. Since the study reported here used only a single low dose of UVB, it is likely that repeated exposures or higher doses would have major consequences for these animals.

This is the first report of an experimental overexpression of a galectin *in vivo*. It is striking that the gain of function (this study) and the loss of function [14] mutations of *galectin-7* have very similar consequences on the maintenance of epidermal homeostasis. Both transgenic mice and null mutant mice display a similar delay in collective cell migration after injury, and they also both have a similar premature apoptotic response following UVB exposure. Taken together, these *in vivo* results argue that an optimal (*wt*) dose of galectin-7 is required to reach a maximal efficiency in skin repair. Different mechanisms can explain such gene balance effects, but one prevalent hypothesis holds that the assembly of multisubunit complexes is sensitive to the precise stoichiometric proportions of individual components [29]. Importantly, defects in adherens junctions, reminiscent of those described here in transgenic mice, have also been found in tail epidermis of *galectin-7* deficient mice (S2 Fig.). Thus, we propose that any stochiometric imbalance in the amount of galectin-7, either too much or too little, may affect the stability of adherens junctions. That galectins can be engaged in multimolecular complexes has already been established [30,31,32]. Interestingly, such gene balance effects could explain some apparently conflicting reports in the field. Depending on the study, the same galectin has been found to be either upregulated or downregulated in apparently similar pathological situations. This so-called « dual » role has been often observed for galectin-1 and galectin-3 [33,34], but it is also the case for galectin-7 [10], possibly even during skin tumorigenesis [9].

In conclusion, it has long been proposed that the level of galectins might play a role in modulating the adhesion/de-adhesion equilibrium in tissue culture cells [35,36]. The present study provides compelling genetic evidence in favor of this balance model acting at the tissue level in mice.

## Supporting Information

S1 Fig Ultrastructural analysis of *wt* and transgenic back epidermis. Representative fields of back skin from *wt* (left) and *tg*_*34*_ (middle and right) mice are shown. Defects of intercellular junctions and basement membrane are visible on low magnification micrographs of *tg*_*34*_ back skin (compare left and middle panel). Gaps (asterisks) between consecutive desmosomes (arrows) are seen in higher magnification of *tg*_*34*_ epidermis, indicating defective adherens junctions. Scale bars: 2μm in left and middle panels, 0.5μm in right panel.
(TIF)

S2 Fig Ultrastructural analysis of *galectin-7* deficient adult tail epidermis. Representative fields of tail skin from *galectin-7* null mutant mice are shown. Left panel: Discontinuities in cell-cell contact (asterisks) are visible on low magnification micrographs of mutant skin. Right panel: Higher magnification revealed abnormal spaces (asterisks) between desmosomes (arrows), indicating defective adherens junctions. Basement membrane is shown with arrowheads. Scale bars: 2.5μm in left panel, 1μm in right panel.
(TIF)
