# Supplementary figures and images for: Overexpression of Galectin-7 in Mouse Epidermis Leads to Loss of Cell Junctions and Defective Skin Repair
Source: PLoS One. 2015 Mar 5;10(3):e0119031. doi: 10.1371/journal.pone.0119031 (PMC4351092; doi:10.1371/journal.pone.0119031)

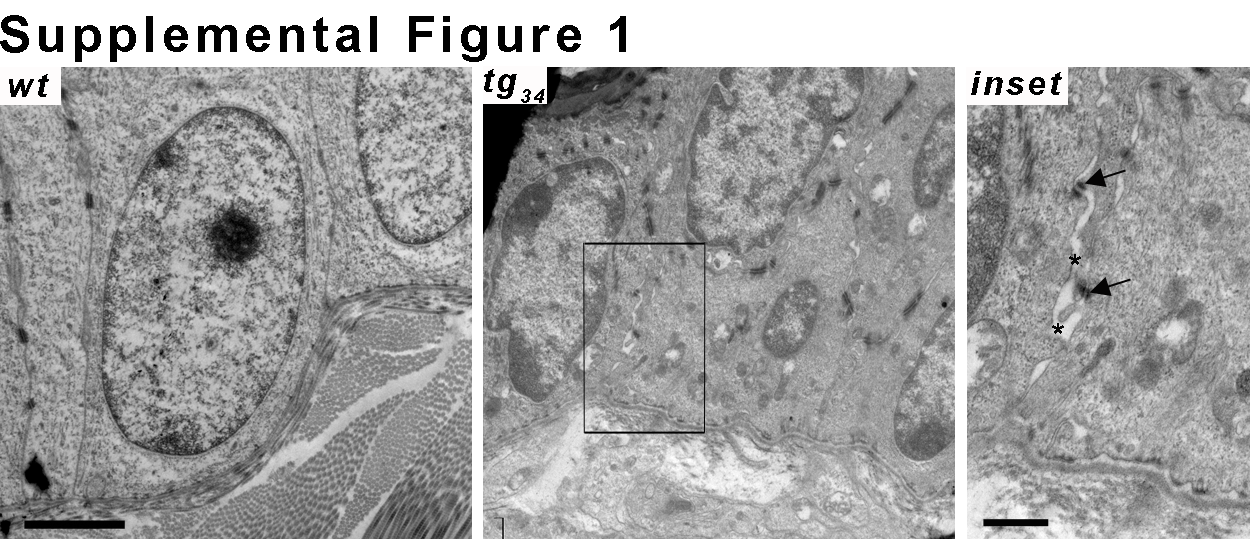

Supplement: S1 Fig — Representative fields of back skin from wt (left) and tg 34 (middle and right) mice are shown. Defects of intercellular junctions and basement membrane are visible on low magnification micrographs of tg 34 back skin (compare left and middle panel). Gaps (asterisks) between consecutive desmosomes (arrows) are seen in higher magnification of tg 34 epidermis, indicating defective adherens junctions. Scale bars: 2μm in left and middle panels, 0.5μm in right panel. (TIF) [file pone.0119031.s001.tif]

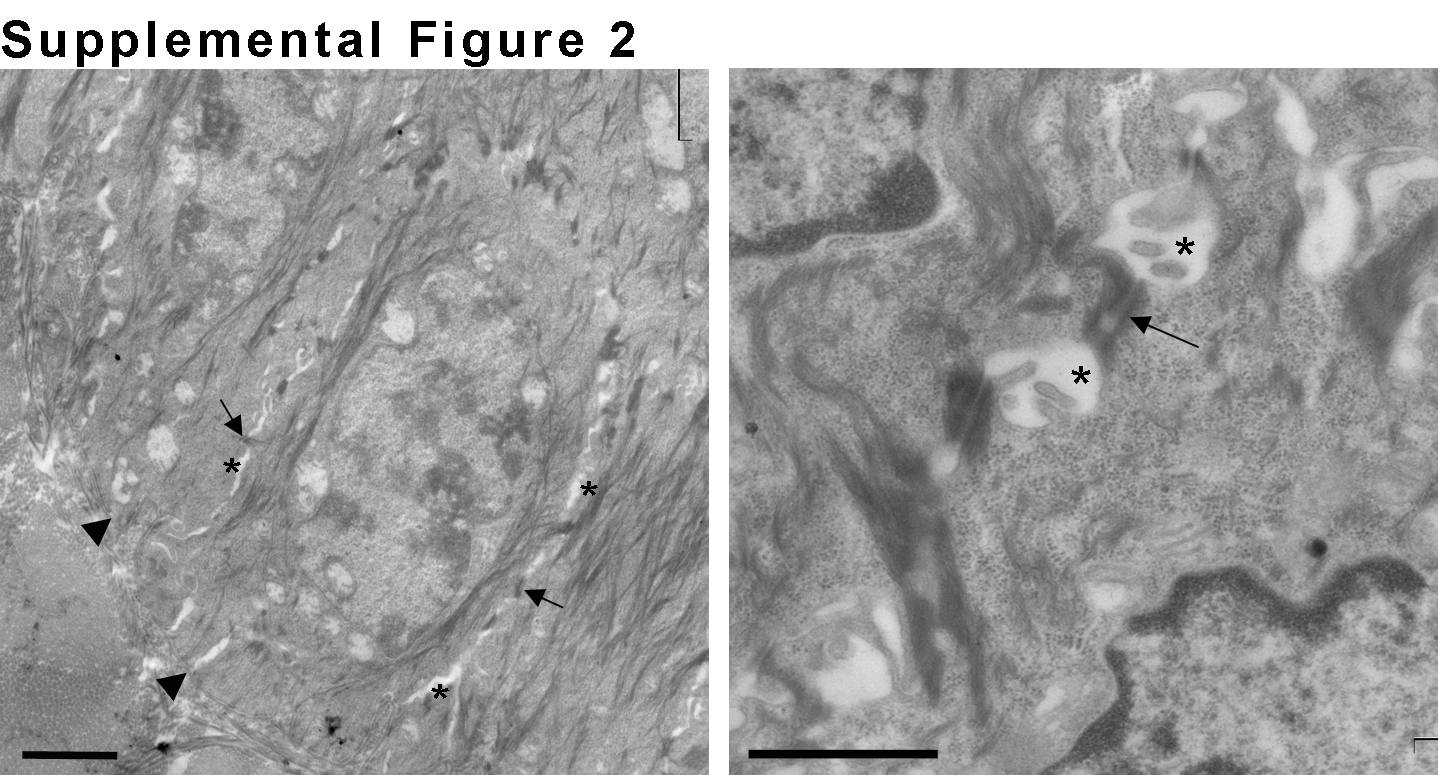

Supplement: S2 Fig — Representative fields of tail skin from galectin-7 null mutant mice are shown. Left panel: Discontinuities in cell-cell contact (asterisks) are visible on low magnification micrographs of mutant skin. Right panel: Higher magnification revealed abnormal spaces (asterisks) between desmosomes (arrows), indicating defective adherens junctions. Basement membrane is shown with arrowheads. Scale bars: 2.5μm in left panel, 1μm in right panel. (TIF) [file pone.0119031.s002.tif]
